# Leprosy stigma in the healthcare setting: Lived experiences of persons affected by leprosy in Niger

**DOI:** 10.1371/journal.pntd.0013584

**Published:** 2025-10-10

**Authors:** Amina Paula Alio, Ibrahim Malam Mamane Sani, Yohanna Abdou, Moussa Gado, Issa Harouna, Bahadir Celiktemur

**Affiliations:** 1 University of Rochester Medical Center, Rochester, New York, United States of America; 2 The Leprosy Mission, Niamey, Niger; 3 Université Abdou Moumouni, Niamey, Niger; 4 Programme Nationale de Lutte contre la Lèpre, Ministère de la Santé, Niamey, Niger; 5 IDEA-Niger, Maradi, Niger; 6 The Leprosy Mission-Great Britain, London, United Kingdom; Federal University of Ceará, Fortaleza, Brazil, BRAZIL

## Abstract

Leprosy-related stigma is deeply rooted in history and permeates every aspect of society, and stigmatization by healthcare professionals is no exception. This paper explores the experiences of persons affected by leprosy with stigma in the healthcare setting throughout their treatment-seeking journey. We conducted one-on-one interviews with 112 individuals who experienced a leprosy diagnosis and underwent treatment. Data was collected in July and August 2022, in nine villages and one urban neighborhood in the region of Maradi, Niger. Over two-thirds of participants experienced some form of visible impairment due to leprosy. Throughout their treatment-seeking journey, healthcare providers were reported to have stigmatized patients by using hurtful language, isolating patients, physically distancing from patients, or refusing to treat. The extent of the stigmatization depended on, first, whether leprosy was recognized or not. Unfortunately, many healthcare providers did not detect the early signs of leprosy, therefore, patients were then not able to obtain treatment prior to physical impairment. Second, the level of stigmatization varied based on the degree and type of visible physical impairments (e.g., missing fingers or toes, limbs, blindness). These negative experiences with healthcare workers made treatment-seeking and adherence to treatment regimen less likely for patients. Our findings confirm the presence of leprosy stigma in the healthcare context in Niger. Increasing leprosy knowledge and addressing stigma among healthcare professionals is key to early diagnosis and treatment of leprosy.

## Author summary

Leprosy is a Neglected Tropical Disease that still exists in Niger. This is in large part due to the stigma associated with the disease. This paper is the result of a study that explored the life experiences of persons affected by Leprosy, focusing on the stigma they experienced while getting treatment in public health centers. Between July and August 2022, we interviewed 112 individuals who experienced a leprosy diagnosis and underwent treatment at some point in their life. We recruited participants from nine villages and one urban neighborhood in the region of Maradi. Participants described experiences where healthcare workers stigmatized them by using hurtful language, isolating them from other patients, physically distancing themselves from them, or refusing to treat them. Many healthcare workers had low knowledge of leprosy and did not detect the early signs. Therefore, patients were then not able to obtain treatment early enough to prevent physical impairment. The level of stigmatization varied based on the type of visible impairments. Because of these negative experiences, patients were then less likely to continue treatment as prescribed. Future interventions should consider improving healthcare workers’ knowledge of leprosy and addressing stigma to ensure timely and better treatment for patients with Leprosy.

## Introduction

Leprosy is a neglected tropical disease (NTD) that has been synonymous with disability and stigmatization throughout history [1,2]. Caused by the bacillus Leprae, leprosy affects the nerves of the skin, causing visible alterations ranging from discoloration in spots, to boils [3]. Loss of sensation is the primary initial symptom distinguishing it from other skin conditions, making initial early diagnosis difficult without a blood test specifically for the bacillus. The disease develops slowly, with an incubation period ranging from 2 to 6 years. It can affect people of all ages and sexes, including children. Leprosy can be cured at any stage of the disease with multi-drug therapy (MDT), and, if detected and treated early, disability can be prevented. When left untreated, the consequences of leprosy are lifelong and negatively affect the physical, social, and emotional well-being of those affected and their families. The sequalae of leprosy include serious impairment of the extremities, including hands, feet and face. Because the loss of sensation is often permanent, even after treatment the person affected by leprosy is destined to a life of self-care to avoid further damage to desensitized skin. This self-care includes protection from the elements and injury. Because of the lack of sensation and not feeling any pain, wounds are often neglected until late stages that result in amputation of a limb. As a result, leprosy has been associated with much stigma not only because of the beliefs associated with its transmission, but the physical impairment and disabilities that ensue [3,4].

Views about leprosy’s causal origins are two-fold. First, there is the fatalistic perception that the disease has both natural and divine origins, and that it is of a punitive nature for acts committed by the individual or a parent. The belief that leprosy is contracted by one who is being punished or cursed by a divine power has been common in multiple religions and examples are recorded in their books (e.g., Bible, Torah, Kuran) [1,4]. In Niger, as in many African countries, leprosy exists and people affected and their offspring are highly stigmatized and shunned from their communities of origin, forcing them to create new communities (often referred to as ‘colonies’) with other persons affected by the disease and their families [5].

Leprosy is no longer considered a Public Health issue, but the disease persists in many developing countries like Niger. The Niger Ministry of Health reports that, out of about 4,000 individuals with skin conditions screened, 689 cases of leprosy were treated, of which 259 were cured in 2022 [6]. Leprosy remains a concern in Niger because new cases continue to be diagnosed (400 cases in 2023), even among children (10% of cases), indicating that leprosy bacteria continue to be transmitted. We expect the real number of cases/ hidden cases to be much higher. Additionally, the physical and social life-long impact on patients and their families render leprosy a greater social issue in Niger.

Consequently, understanding the manifestations and drivers of leprosy stigma, as described by the World Health Organization (WHO) stigma framework [7], is key to informing interventions to reduce stigma and improve the lives of those affected and their communities. In the case of leprosy, early detection and adherence to treatment is key to preventing physical impairment (e.g., missing fingers, toes, limbs, disfigurement, blindness) and associated disability (experiences with environmental and social barriers/structures).[8] Stigma is a concept embodying elements of labeling, stereotyping, ‘othering’, separating, status loss and discrimination occurring within a situation of power [9]. Stigmatization by family and community members, as well as by healthcare providers is the primary barrier to patients accessing screening services and treatment. Studies on community stigma in African countries are multiple, however, very few have focused on the problem of stigma by healthcare providers. The few studies that describe stigmatization by healthcare providers highlight the need to better understand the phenomenon and to address the problem [10–12]. Mirroring their society, healthcare providers have been known to discriminate against patients with leprosy or those living with the sequelae. A study in Ghana recognized that healthcare providers were afraid of leprosy and therefore avoided patients who had the disease because of the visible sequelae [13]. A study in Nepal, although it does not directly address discrimination by healthcare providers, described the effects of stigma on healthcare seeking behaviors [14]. Stigma was found to affect where patients sought treatment, with many of them opting to obtain treatment from spiritual or traditional healers, which delays their treatment and increases chances of resulting impairments and disability.

### Objectives

Despite advancements in knowledge about the disease and its treatment, leprosy stigma persists in society, and particularly in healthcare settings [10–14]. Misconceptions about the disease and its transmission still abound, leading to discrimination and prejudice against leprosy patients and patients living with the sequelae of the disease and needing treatment for co-morbidities.

Given the gap in the literature about the topic in the context of Niger, this paper presents results of a qualitative study that used a descriptive approach [15] to exploring the experiences of individuals affected by leprosy in Niger. It sheds light on the stigmatization faced by these individuals throughout their treatment seeking journey, particularly within the healthcare setting.

## Methods

### Ethics statement

The study was approved by the Ministère national de l’enseignement supérieur, de la recherche et de l’innovation technique in Niamey, Niger, and by the University of Rochester Research Subject Review Board, New York, USA. Formal verbal consent was obtained from each participant.

### Participants

Participants were recruited via the Maradi regional division of IDEA (Intégration, Dignité, Economie en Avant), an international organization that brings together persons and communities affected by disability, including those affected by leprosy. IDEA is focused on the social integration and economic advancement of persons affected by disability and has chapters across countries globally. IDEA Niger was instrumental as a community partner and stakeholder in this study.

### Setting

In July and August 2022, we conducted individual interviews with 112 participants with a diagnosis of leprosy (past or present) in 10 communities (nine villages and one urban neighborhood) composed of persons affected by leprosy and their children for multiple generations. These sites are in the region of Maradi, one of seven regions in Niger, situated in the south-central part of the country and bordering the country of Nigeria. It is the most densely populated region in Niger, with approximately five million inhabitants (2020 estimate). The region of Maradi was selected because it has the largest number of communities of persons affected by leprosy in the country. The region also is the location of the Danja Hospital (in the town of Danja), the largest of two [2] hospitals with a specialized leprosy treatment center in the country. The settlements of persons affected by leprosy are in the southern part of the region of Maradi, and accessible to the Danja Hospital where patients were treated. We selected 10 out of 15 official settlements based on geographic accessibility. Important for this study, in these settlements are individuals from several regions of Niger who came to Danja Hospital for treatment and remained.

### Data collection

We used a purposive criterion sampling for recruitment. IDEA Maradi discussed the study with community leaders from all 10 communities, after which announcements were made at community gatherings, via telephone messaging, word of mouth and WhatsApp. Participants meeting the criteria of having been diagnosed with leprosy at any point in their lives and who were 18 years or older, were invited to come to a specific community center or square at a specific date and time. One-on-one interviews were conducted in a secluded setting or room to ensure privacy. Interviews were audio recorded with the written informed consent of the participants. The interview guide, pilot tested in Hausa with members of IDEA, was semi-structured in order to maintain consistency between the 5 trained interviewers. Structured questions served to collect information about demographic characteristics and the sequelae of leprosy (i.e., impairments and disabilities), while open-ended questions focused on participants’ experiences from the time of diagnosis to the present. Probes focused on manifestation of stigma in the healthcare setting and the drivers of stigma. Interviews lasted on average 45 minutes (range of 35–70 minutes). Interviews were conducted in Hausa, the primary language of the region, and known to all the interviewers. Each interviewee received 2,000 CFA francs (approximately 4 USD) for their participation. The sum was determined by IDEA and deemed appropriate for participants’ time and transportation costs.

### Data analyses

Interview audios were transcribed by the interviewers using direct translation from oral Hausa into written French. Transcripts were verified for accuracy by the project advisory team of stakeholders (including persons affected by leprosy) to discuss the accurate translation of certain local concepts. Transcripts were uploaded into Dedoose and coded using a descriptive, narrative analysis approach [15] within the context of the phenomenon of interest, the treatment-seeking stories of persons affected by leprosy. The narrative focused on describing common experiences in the journey to healing. Multiple coders were used, and disagreements discussed until consensus was reached. Members of IDEA who themselves are affected by leprosy participated in discussions to validate or correct researchers’ understanding of the data and its interpretation.

### Researcher positionality

All co-authors were investigators in the study and represent backgrounds in Anthropology, Public Health, Sociology, Infectious Diseases, Dermatology, community leadership and advocacy, and lived experience with leprosy. Five of the six co-authors are Nigeriens and fluent in French and Hausa. Two of the Nigerien co-authors are persons affected by leprosy from one of the study sites. The fifth co-author is not from Niger but has done work in the country in the past and, for this study, participated in the design, analysis, and write-up of study results. The five Nigerien authors participated in all aspects of the study, including conducting the interviews. To avoid bias or conflict, they did not conduct interviews in their own communities. The Nigerien co-authors’ ethnic and national background and firsthand experiences with leprosy may have influenced interpretation of the data. To avoid speaking for the data, they made efforts to bracket biases and assumptions. The sixth author was instrumental in raising questions about clarity and underlying assumptions.

## Results

Of the 112 persons affected by leprosy interviewed, 47 were female (42%). Though most over the age of 70 were not sure of their exact age, participants were adults, they estimated being between 35 to over 70. Most (64%) were diagnosed late or where not initially adherent to treatment and had grade 2 disabilities associated with leprosy [3], including damage to the feet (n = 22), hands (n = 33), face (n = 15), both hands and feet (n = 2), and other impairments (n = 17) like the loss of a limb, for example. Almost all had no formal schooling (95%) but 30% had attended literacy classes as part of community activities by non-governmental organizations (NGOs) and community-based organizations (CBOs). Most participants were married (71%) or widows/widowers (20%). They had an average of 4.7 children each, ranging from no children to 19. Participants made ends meet primarily income-generating activities including farming, breeding animals, small commerce/business, or other manual labor. Over a third (38%) reported begging in the streets as an important source of income (Table 1).

**Table 1:** Participants’ characteristics (N = 112).

Characteristic	Value	%^a^
Age		
35 – 55	28	25%
56 – 69	35	31%
≥ 70	49	44%
Sex		
Female	**47**	42%
Male	**65**	58%
Disability		
Grades 0 or 1	**23**	**21%**
Grade 2	**72**	**64%**
Unknown	**17**	15%
Education		
No formal education	**106**	95%
Primary school	**2**	2%
Not known	**4**	4%
Marital Status		
Never married	4	4%
Married	79	71%
Divorced/Widowed	27	24%
Unknown	2	2%
Number of children		
Mean	**4.7 children**	
Range	**0 – 19 children**	
Livelihood^b^		
Income Generating Activities	81	73%
Begging to generate income	43	38%
Unable to work	4	4%

This table presents the demographic characteristics of study participants.

^a^Totals may not add up to 100% due to rounding.

^b^Totals may not add up to 100% because participants could select more than one response.

### Diagnosis of leprosy

The narratives followed two primary paths: those who were diagnosed and treated in the early stages of the disease, and those who were not and therefore experienced physical impairment and/or disabilities. Most noticed initial symptoms like spots on their skin and immediately sought care at a clinic or from a traditional healer. Unfortunately, for the majority, leprosy was not immediately diagnosed, and they were treated for other skin conditions. When the symptoms became worse, they sought care from traditional healers to no avail and then turned to formal medical clinics or hospitals. Because the symptoms had become worse, leprosy became more recognizable and was finally diagnosed. For most, they were diagnosed with leprosy as adolescents or young adults, although the initial symptoms indicate they had had it for a while.

For about a third of the participants, the diagnosis occurred early because of someone in their social circle recognizing the symptoms because they had experienced them or knew someone who had. A male participant who was diagnosed as a child remembers: *“I was visiting my uncle, and he told me that I needed to go to this special hospital to be treated for my skin condition because it could be leprosy. So, they took me to the Danja Hospital.” (#9, Male)* Those who were diagnosed early or eventually all ended up at Danja Hospital where they were diagnosed with leprosy and treated.

The reactions to the diagnosis were similar across participants. They felt it was “*worse than a death sentence*,” (#23, Male) because they would be living with a disease that would change the course of their lives. All ended up leaving their home and community of origin, for several reasons, the primary being:

- rejection by their family or community: “*When my parents found out, they were afraid and sent me away. But everywhere I went people treated me badly until someone told me about Danja*.” (#96, Male)
- fear of rejection by their family and community: “*I knew if people find out what I have they will be afraid of me or think something bad about me*.” (#2, Female)
- fear of the social consequences on their family: “*I was married and didn’t want my husband and children to suffer because of me, so I left my husband and 2-year-old child. I left my home to come to Danja where I started a new life.*” (#24, Female)
- the desire to find treatment at the Danja Hospital: *“My parents took me to Danja because they knew someone who was treated there. They left me with them and returned home*.” (#1, Male)

### Seeking treatment

All participants described doing all they could to treat their newly diagnosed condition of leprosy. To them and their parents (for those who were children), that meant seeking both spiritual/traditional and modern medicine. Because leprosy is seen as having an important spiritual aspect, medical treatment for the physical symptoms was not considered sufficient: the perception was that all aspects of the disease needed to be addressed for healing. Once diagnosed with leprosy, going to the spiritual, traditional healer was purposeful and they finally had a specific reason for seeking healing. This was done parallel to the treatment at the Danja Hospital for most. – mainly those who were younger at the time - quickly abandoned the harsh traditional treatment that consisted of incantations and very bitter potions to drink and specially made ointments for the affected areas of the skin. One man describes his experience and as a child: *“It was so bitter! I didn’t want to drink it anymore. It made my stomach sick, and I would vomit. So, I would cry and refuse even when people would tell me I needed it.” (#45, Male)* Of note is that participants described positive experiences with the healers themselves, if not the treatment. The traditional healers did not stigmatize the patients that came to them with a diagnosis of leprosy. They did not fear them or reject them, as it seemed to have been a spiritual matter that they were equipped and ready to deal with.

Parallel to the traditional treatment, many also sought medical treatment. Unfortunately for over 60% of our sample, the treatment came after the visible physical impairments and disabilities. The few who sought treatment in local health centers were put on treatment or referred to the few regional hospitals with medication for leprosy. However, their experiences were not as positive as with the traditional healers. Some talked about clinicians refusing to treat them in a manner that was full of disgust or pity for their plight. The feelings of pity were, as the patients perceived, because they were suffering the divine or spiritual wrath for something they of their family did. *“They sent me to the hospital in Tahoua; and there, they felt sorry for my condition, saying ‘It is God’s doing.’” (#36, Female)* They perceived this attitude of pity as hurtful and bringing up feelings of shame for having leprosy. Others described their interactions with healthcare workers as very negative, including being isolated from other patients, or the workers keeping a distance from them for fear of contagion. “*They didn’t want to touch me. They were afraid to catch what I had*.” (#12, Male) These reactions of pity, fear and disgust were common among the study participants’ experience with the healthcare workers of all levels, from the clinicians to the staff. The health centers that treated them made it difficult to return regularly for their medication. *“They gave me pills for 2 months and told me to come back, but I didn’t. I continued with my traditional treatment after the pills finished.”* When asked why he did not return, the man explained “*I just didn’t. I didn’t want to feel ashamed.” (#47, Male)*

Eventually, all the patients ended up at the Danja referral hospital for leprosy, the largest one in the country. Danja Hospital, located in the far south-east of the country, includes a primary focus on the treatment of persons with leprosy. The patients were either referred by someone they knew who recognized the signs of leprosy, or by a clinician who diagnosed them or suspected a leprosy diagnosis but was unable to confirm. The patients described the treatment they received in Danja Hospital as holistic, dealing with the physical, emotional, social, and spiritual aspects. “*They gave me medication and allowed me to stay there until my treatment was finished. I was there for many months*” (#73, Male). “*At Danja I found many others like me. Some had just arrived; some had been there for a while.”(# 85, Female) “They explained to me my condition and how to take care of my feet.” (#4, Male) “They helped me with my emotions. We could talk to them or to other people like me anytime.” (#101, Female) “I was feeling bad, but everybody was so nice and accepting of me.” (#15, Female)* At Danja Hospital, they found a community of people who shared similar experiences and staff who cared for their well-being beyond leprosy. “*The [Danja staff] really took their time and told me I could stay there until I was healed. So, my family left me there to go back to our home region. I’m still here after many years. I married and had children and I’m still here in this community.” (#7, Male)*

### Living with the sequelae

As they live with the sequelae of leprosy, participants with grade 2 disabilities (visible impairments or deformities) describe avoiding health centers outside of Danja to evade being stigmatized and discriminated against by healthcare workers. Though cured of the disease, they must suffer the long-term social effects of leprosy, including fear, disgust, and pity. *“We only go to Danja when we’re sick with anything, or when I have a sore that won’t heal.”(109, Male) “Danja is our hospital. Even if I go to the clinic here, when they see we [have leprosy] they automatically tell us to go to Danja. Even if we finished our leprosy treatment.” (68, Female)*

Meeting a community of people sharing the same illness and social fate, the patients intermarried, created families, and established multiple settlements that are now villages and an urban neighborhood. These communities of persons affected by leprosy, including patients, former patients and their offspring have become a safe haven for the newly diagnosed who come from the various regions of Niger and northern Nigeria for treatment.

## Discussion

That leprosy stigma exists in healthcare settings has been established in the literature [10,16,17], and our study is no exception. Healthcare workers’ negative attitudes toward leprosy and persons affected have been identified in multiple countries, including India [18], Thailand [19,20], Ethiopia [21], and Guyana [22]. In our study, the physical manifestations of leprosy evoked fear and/or revulsion among healthcare workers. A review of leprosy stigma in healthcare settings [16] highlighted studies indicating that health workers’ reactions to leprosy are fear of contagion, fear of associated infirmities and disgust, which result in a reluctance to treat patients affected by leprosy [20,21]. In our study, these feelings also manifested as reluctance to provide care, segregation of leprosy patients from other patients, or even refusal to touch or examine them.

Stigma towards persons affected by leprosy can have detrimental effects on patients’ mental health and well-being. Consequences of stigma from healthcare workers include perpetuation and reinforcement of social isolation, and reluctance of patients to obtain and/or adhere to treatment [18,19,23,24]. For our respondents, being subjected to discrimination and prejudice from healthcare providers led to feelings of shame, embarrassment, and low self-worth. This, in turn, deterred them from seeking a timely diagnosis and medical treatment. The delayed diagnosis, delayed treatment and non-adherence resulted in permanent damage to their skin, face, hands, and/or feet.

Research findings indicate that higher knowledge and awareness of leprosy among healthcare professionals is associated with more positive attitudes and less stigma [16,25,26]. Our findings indicate a lack of accurate knowledge about the disease among healthcare professionals, similarly to Ethiopia where a study [21] reported 86% of healthcare workers had low overall knowledge of leprosy and only 18% diagnosed leprosy correctly, and South Africa [27] where only 15% of healthcare workers felt they had sufficient knowledge to treat leprosy. Most of our participants who first went to clinics or hospitals outside of the main regional hospitals were not immediately diagnosed when the symptoms were limited to the skin, when disability could have been prevented. About a third were lucky enough to have someone with personal experience with the disease to refer them to Danja Hospital for accurate diagnosis and treatment. This highlights the importance of training clinicians in how to recognize the early signs of leprosy and how to treat it, given the medication is at no cost to the patient and available in Niger. This has been recognized in the country and is one of the goals of the National Leprosy Control Program.[6] Early diagnosis and treatment would be lifechanging for persons affect by leprosy, as they would not suffer from the physical sequalae, and the stigma towards them would be greatly diminished. The participants who had no visible infirmities were better able to integrate society outside of their communities, although they lived with the fear of being found out. Should it be known that they had had leprosy, there would be reticence to have them integrated fully and they would be stigmatized in several ways, ranging from mockery and name calling to refusal to give their daughter’s hand in marriage. Such stigmatization is common in sub-Saharan Africa, as indicated in studies from Nigeria [28], Togo [29] and Côte d’Ivoire [30], for example, all of which emphasize persistent social stigma related to leprosy.

In addition to healthcare providers, stigma within healthcare settings can also be perpetuated by institutional policies and practices. Historically, persons affected by leprosy have been isolated socially in “colonies” [31,32] and institutionally in leprosaria [33,34], with policy changes occurring as late as the 1990’s in some countries [35,36]. Having separate leprosy hospitals, while intended for improved patient care and disease control, can reinforce social isolation of persons affected, perpetuating fear and discrimination [37]. For example, having a large hospital like the one in Danja for the care of persons with leprosy, while being a place for specialized and holistic care, may have the unintended consequence of reinforcing the idea of segregation and otherness. This could be seen in our findings: the patients found safety in perpetuating the idea that Danja is their hospital, a place they feel welcomed. For these patients, Danja has become a safe place for healing and overall well-being, showing adaptation to the lifelong and intergenerational stigmatization of leprosy. At the same time, villages such as Danja have become stigmatized by the larger society, with anyone from the area treated as a person affected by leprosy. To remedy institutional issues and considering the reduced incidence of leprosy worldwide, properly planned integration of leprosy services has been the goal long suggested by the WHO.[38,39]

Addressing leprosy stigma in healthcare settings requires a multi-faceted approach that includes increasing healthcare providers’ knowledge of leprosy, addressing leprosy stigma among healthcare workers, and ensuring institutional policies emphasize patients’ right to care [11,40]. First and foremost, healthcare providers need to be educated about leprosy, its causes, transmission, and treatment. Training programs should focus on dispelling myths and misconceptions about the disease and promoting empathy and compassion towards patients. Additionally, healthcare facilities should implement policies that promote inclusivity and non-discrimination, such as integrating leprosy care into general healthcare services and ensuring equal access to treatment for all patients at all stages of the disease.

Training of healthcare provider should also include how to address stigma at the different points of the treatment-seeking journey of patients. For example, when diagnosing leprosy, healthcare workers can be trained to provide counseling (similar to HIV diagnosis) [41] to address the fear of the disease and its social consequences while emphasizing the importance of adherence to treatment regimen for preventing impairment. For those diagnosed late, healthcare workers can be sure to address any social/environmental factors causing disability as a result of their impairment, while emphasizing the importance of adherence to treatment regimen and self-care needed to prevent further physical damage. At any point in the patient’s treatment process, most important is for them to model acceptance and compassion.

### Limitations

This study is limited in that it only represents the views of patients about their experiences in healthcare centers, as was the goal of this study. We do not represent the perspective of healthcare professionals. Future research may focus on measuring stigma among healthcare professionals to better understand their views about the disease and the people affected. Nevertheless, understanding the experiences of patients is most important to identify the existence of stigma and call for interventions to address stigma in the healthcare context, especially since it affects patient treatment-seeking behaviors.

## Conclusions

Leprosy stigma in healthcare settings persists due to a combination of lack of knowledge and fear. Addressing this stigma requires concerted efforts to educate healthcare providers, reform institutional practices, and engage communities in promoting acceptance and inclusion. Traditional/spiritual healers should be considered when planning interventions for leprosy screening, as they can play a significant role in helping to identify leprosy in its early stages. Training of healthcare providers is also key to helping combat stigma and ensuring that leprosy is diagnosed and treated early, sparing patients lifelong consequences of impairment and resulting disabilities. As stigma among healthcare providers stems partly from fear of contagion, an increase in knowledge about the fact that leprosy is one of the least contagious infectious diseases would help alleviate these fears. The example of the Danja Hospital staff’s treatment of leprosy patients is evidence that trained healthcare workers and an approach to care that includes addressing the social consequences of leprosy can lead to better detection, treatment, and the overall well-being of patients. By challenging stigma and fostering empathy and understanding among healthcare providers, we can ensure that all patients, including those affected by leprosy, receive equitable healthcare and support.

## Supporting information

S1 Table Table of themes by time of diagnosis. This table presents the major codes and themes that emerged from analsyses of participant interviews. These major codes and themes are displayed in three columns. The first colums represent the participants diagnosed early in the disease progression prior to any infirmities, and the last column displays the major codes and themes found among participants diagnosed at a late stage who suffered impairments or disability. The middle column presents the themes common among both groups.
(PDF)

S1 Fig Flowchart of themes by time of diagnosis and sequence of events. This flowchart displays the themes of the 2 groups and the sequence of events as experienced by participants. The two groups are the participants diagnosed early in the disease progression (prior to any infirmities) on the left, and thoses diagnosed at a late stage who suffered impairments or disability (far right). In the middle are the themes common to both groups.
(PDF)

S1 File Questionnaire. **Interview Questionnaire.** This document lists the questions asked of the participants, including demographic characteristics (e.g., age, sex) and questions regarding their experiences with healthcare from diagnosis to treatment.
(DOCX)
