# Supplementary material for: Leprosy stigma in the healthcare setting: Lived experiences of persons affected by leprosy in Niger
Source: PLoS Negl Trop Dis. 2025 Oct 10;19(10):e0013584. doi: 10.1371/journal.pntd.0013584 (PMC12527163; doi:10.1371/journal.pntd.0013584)
Supplement: S1 Table — This table presents the major codes and themes that emerged from analsyses of participant interviews. These major codes and themes are displayed in three columns. The first colums represent the participants diagnosed early in the disease progression prior to any infirmities, and the last column displays the major codes and themes found among participants diagnosed at a late stage who suffered impairments or disability. The middle column presents the themes common among both groups. (PDF) [file pntd.0013584.s001.pdf]

Themes by time of diagnosis.

Participants Diagnosed Early (36%)	Common Themes	Participants Diagnosed post-sequelae (64%)
Family member **recognized potential signs of Leprosy**.<br>Family member **suggested Danja Hospital**	**Experienced symptoms:**<br>-Spots on skin<br>-Rash	Sought Care at Clinics<br>Traditional Healers
**Went to Danja**<br>With family support		**Misdiagnosed**<br>Treated for other skin conditions
		**Experienced physical sequelae of Leprosy**:<br>Loss of or deformity in toes/feet and/or fingers/hands, facial deformities
**Diagnosed with Leprosy at Danja**<br>Remained in Danja for Treatment	**Reaction to Diagnosis of Leprosy:**<br>-"Worse than death"<br>-Feared family/community rejection/shame<br>-Feared repercussion on family	**Leprosy Recognized & Diagnosed in Clinics:**<br>- **Experienced Stigma & Discrimination** from clinical staff; clinic Staff: didn't want to touch them; isolated them; refused to treat them; sent them away; spoke to them with pity or disgust.<br>**Experienced Community-Level Stigma:** mockery, insults, low-expectation, accused of being cursed<br>-**Had to leave family/community**
	**Sought treatment from Traditional Healers:**<br>-Addressed Spiritual Aspect<br>-Harsh medicine<br>-Positive treatment: Well received, well treated	Referred to Danja by clinic staff or others in network
	**Sought Treatment at Danja:**<br>-Well received & well treated by staff<br>-Addressed emotional aspects<br>-Addressed spiritual component<br>-Addressed social aspect<br>-Addressed rehabilitation.<br>-Found social network/community of people affected by Leprosy	
	**Remained in the region** and joined local communities of people affected by Leprosy:<br>-**Established in Danja:** married, had children	
	-**Continue to use Danja Hospital** for all medical needs<br>-**Avoid other clinics/hospitals to avoid stigma and discrimination** by healthcare providers and staff	
