# Supplementary figures and images for: Leprosy stigma in the healthcare setting: Lived experiences of persons affected by leprosy in Niger
Source: PLoS Negl Trop Dis. 2025 Oct 10;19(10):e0013584. doi: 10.1371/journal.pntd.0013584 (PMC12527163; doi:10.1371/journal.pntd.0013584)

## Themes by time of diagnosis and sequence of events.

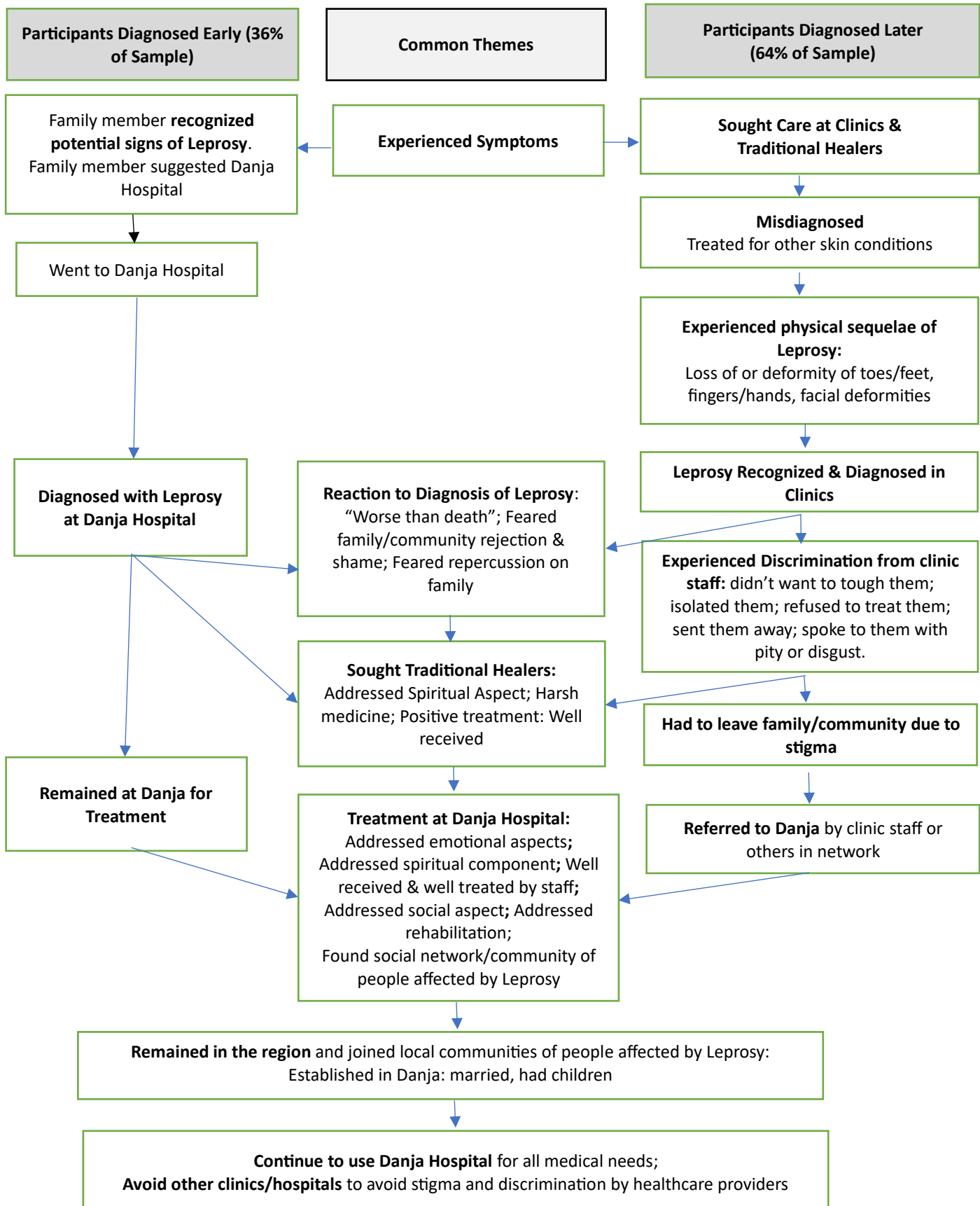

Supplement: S1 Fig — This flowchart displays the themes of the 2 groups and the sequence of events as experienced by participants. The two groups are the participants diagnosed early in the disease progression (prior to any infirmities) on the left, and thoses diagnosed at a late stage who suffered impairments or disability (far right). In the middle are the themes common to both groups. (PDF) [file pntd.0013584.s002.pdf]
