# Supplementary material for: Leprosy stigma in the healthcare setting: Lived experiences of persons affected by leprosy in Niger
Source: PLoS Negl Trop Dis. 2025 Oct 10;19(10):e0013584. doi: 10.1371/journal.pntd.0013584 (PMC12527163; doi:10.1371/journal.pntd.0013584)
Supplement: S1 File — Interview Questionnaire. This document lists the questions asked of the participants, including demographic characteristics (e.g., age, sex) and questions regarding their experiences with healthcare from diagnosis to treatment. (DOCX) [file pntd.0013584.s003.docx]

**Leprosy Stigma Reduction Intervention (LSRI)
Niger, 2022-2025**

**FRENCH Version**

**QUESTIONNAIRE (Personnes affectées par la lèpre)**

**« Manifestation du stigma contre les personnes affectées par la lèpre dans les structures de santé au Niger »**

Numéro d’identification: (initiales de l’enquêteur et N^o^ de l’enquête) _________________

Date : ___________________________

Région : ______________________________________

Décrivez les séquelles visibles (ou N/A): __________________________________________________________

___________________________________________________________________________________________

1. Age : ____________
2. Lieu de provenance : ________________________________
3. Sexe :
   - Féminin
   - Masculin

- Autre

1. Niveau d’études :

- Jamais été à l’école
- École Primaire
- Secondaire
- Formation Professionnelle
- Supérieure
- École Coranique
- Franco-Arabe : Primaire
- Franco-Arabe : Secondaire

1. Situation matrimoniale :

- Jamais marié(e)
- Marié(e) : Nombre de femmes : _____ Nombre de coépouses : ________
- Divorcé(e)
- Répudiée
- La/les femmes a/ont quitté
- Veuf/Veuves
- Autre : _________________________________________________

1. Activités génératrices de revenu :

- Agriculture
- Élevage
- Petit commerce
- Mendicité
- Autre : _________________________________________________

**QUESTIONS QUALITATIVES :**

1. Nous vous prions de nous raconter votre histoire commençant par votre diagnostic de la lèpre.
   1. Qu’est-ce qui vous a poussé à aller vous faire examiner? [Quels symptômes aviez-vous?]
   2. Quand et comment avez-vous été diagnostiqué ?
   3. Dans quelle clinique/centre de santé?
   4. Comment vous ont-ils accueilli?
   5. Comment vous-ont-ils parlé du diagnostic? De la Lèpre? De son traitement?
2. Quelle a été votre réaction?
3. Qu’a été la réaction de votre famille? Votre communauté?
4. Avez-vous suivi un traitement? Quand? Où avez-vous chercher un traitement pour cette maladie?
   1. Comment avez-vous choisi ce [centre de santé/guérisseur traditionnel]? Quelles étaient vos motivations?
   2. Quelle a été la réaction du [personnel de santé/guérisseur traditionnel]? Comment vous-ont-ils traité?
   3. [*Si guérisseur traditionnel seulement, posez ces questions*:]
      1. Quand est-ce que vous avez décidé d’aller à la clinique/hôpital? Quelle était votre motivation?
      2. Comment vous ont-ils accueilli?
      3. Comment ont-ils réagi à votre diagnostic de lèpre?
      4. Comment vous-ont-ils traité?
      5. Comment était le traitement?
      6. Comment était votre expérience avec cet hôpital/cette clinique/ce guérisseur?
5. Quand est-ce que vous avez décidé de venir à l’hôpital de Danja? Qu’est-ce qui vous a motivé?
   - 1. Comment vous ont-ils accueilli?
     2. Comment ont-ils réagi à votre diagnostic de lèpre?
     3. Comment vous-ont-ils traité?
     4. Comment était le traitement?
     5. Comment était votre expérience avec cet hôpital?
6. Qu’est-ce qui vous a poussé à rester dans cette région?
   1. Comment est votre vie maintenant? [La famille? Le gagne-pain? Les activités? La vie communautaire?]
7. Avez-vous autre chose à ajouter?

**ENGLISH Version**

**QUESTIONNAIRE (People affected by leprosy)**

**“Manifestation of stigma against people affected by leprosy in health structures in Niger”**

Identification number: (investigator's initials and survey number) ^_________________^

Date: ___________________________

Region: ______________________________________

Describe the visible after-effects (or N/A): __________________________________________________________

___________________________________________________________________________________________

1. Age: ____________
2. Place of origin: ________________________________
3. Gender:
   - Female
   - Male

- Other

1. Level of education:

- Never been to school
- Primary school
- Secondary
- Vocational training
- Superior
- Koranic School
- Franco-Arab: Primary
- Franco-Arabic: Secondary

1. Marital status:

- Never married
- Married: Number of wives: _____ Number of co-wives: ________
- Divorcee)
- Repudiated
- The woman(s) left
- Widow/Widowers
- Other: _________________________________________________

1. Income generating activities:

- Agriculture
- Breeding
- Small business
- Begging
- Other: _________________________________________________

**QUALITATIVE QUESTIONS:**

1. Please tell us your story, beginning with your diagnosis of leprosy.
   1. What prompted you to go get checked out? [What symptoms did you have?]
   2. When and how were you diagnosed?
   3. In which clinic/health center?
   4. How did they welcome you?
   5. How did they tell you about the diagnosis? About leprosy? About its treatment?
2. What was your reaction?
3. What was the reaction of your family? Your community?
4. Did you receive treatment? When? Where did you seek treatment for this disease?
   1. How did you choose this [health center/traditional healer]? What were your motivations?
   2. What was the reaction of the [health worker/traditional healer]? How did they treat you?
   3. [ *If traditional healer only, ask these questions*:]
      1. When did you decide to go to the clinic/hospital? What was your motivation?
      2. How did they welcome you?
      3. How did they react to your leprosy diagnosis?
      4. How did they treat you?
      5. How was the treatment?
      6. How was your experience with this hospital/clinic/healer?
5. When did you decide to come to Danja Hospital? What motivated you?
   - 1. How did they welcome you?
     2. How did they react to your leprosy diagnosis?
     3. How did they treat you?
     4. How was the treatment?
     5. How was your experience with this hospital?
6. What made you want to stay in this region?
   1. What is your life like now? [Family? Livelihood? Activities? Community life?]
7. Do you have anything else to add?
